# Treating Infected Root-Canals with Kalium and Natrium

**Published:** 1894-04

**Authors:** Emil Schreier

**Affiliations:** Vienna, in Columbian Congress and Discussion.


ARTICLE III.

### TREATING INFECTED ROOT-CANALS WITH KALIUM AND NATRIUM.

BY DR. EMIL SCHREIER, VIENNA, IN COLUMBIAN CONGRESS AND DISCUSSION.

When a tooth with gangrenous or necrotic pulp comes under treatment, the dentist is confronted with the task of removing as far as possible a gelatinous, slightly consistent mass from a capillary tube, and, this having been accomplished, to introduce into the same canal an antiseptic for purposes of disinfection. You are all aware how much time, patience, and skill are necessary for this operation. The average dentist has enough trouble in many cases in simply probing the canal with a delicate needle, not to speak of cleansing, and much less filling; he is accordingly compelled to leave out of consideration any thought of saving the tooth. Such cleansing, however, is unnecessary, if it be possible to convert the septic contents of the canal into an aseptic condition, and the operation is much simplified if it be possible to effect the transformation by the simple introduction of a nerve-needle.

My method seeks to fill the first indication by a chemical decomposition of the putrescent contents, in which the root-canal serves as a test-tube; the second indication is fulfilled in the development of a substance which is readily taken up by a nerve-needle, and sufficiently adhesive for introduction into the canal. This substance consists of kalium and natrium in a metallic state. I pierce its paraffin stopper with a nerve-needle chosen at random. You observe a delicate deposit resembling quicksilver on the needle. I now dip the needle in a glass of water; the needle describes a fiery tract therein. In the root-canal in question there exists a putrescent mass. This consists of water and the decomposition product of albumen, the latter consisting especially of fats and fatty acids.

These substances have been formed by the influence of bacteria, and serve as a culture-medium for the various species contained therein. If I now introduce my preparation into the canal with the needle, decomposition of the watery contents will occur, with development of a considerable amount of heat. Potassium and sodium hydroxide are formed, which, in combination with the fat of the pulp, form soap. The characteristic gangrenous odor is accordingly changed into a well-marked soapy smell. A portion of the alkalies possess the well-known property of rendering albuminus substances soluble. Thus any remains of tissue adherent to the walls of the canal are dissolved, the latter become macerated, and access to the dentine canaliculi is possible sooner than can be effected by any other method. Destruction of the organic contents of these canals is now possible. In consequence of such destruction the disagreeable discoloration, which too frequently occurs, will be absent, and the lime-salts of the tooth proper are in no wise injuriously affected by the treatment.

The introduction of the potassium and sodium has the additional effect of destroying the bacterio, partly by the heat produced, and partly by the new products formed. The contents of the canal have been transformed into a sterile and probably antiseptic mass, and thus the development of new colonies of bacteria is prevented. Everything has thus been accomplished which precedes permanent filling of the tooth.

### DISCUSSION.

The question was asked if these chemically prepared needles vary in size and can be used in extremely small roots, and also if it is a painful operation.

Dr. Schreier.—The operation is not painful at all, because the teeth are not sensitive.

A Member.—Can you regulate the heat by the strength of the chemical preparation which you use?

Dr. Schreier.—No; not at all. I can only regulate it by varying the amount of the preparation. If you take too much of the preparation it is dangerous, because it might cause an explosion.

Dr. Callahan.—I have been using this preparation for some time; the heat evolved is perfectly harmless. I have tried several experiments, and have put water in the tooth and used a larger quantity of the preparation to see if it would cause any damage, but I don't think I ever injured a tooth in any way by doing so.

Dr. Custer.—What is your after-treatment?

Dr. Schreier.—I beg you to bear in mind that I do not recommend any further treatment, because every man may proceed with the treatment as he is accustomed to. Some fill the canal with cotton, others with oxiphosphate, while others do not fill it at all. I only wish to give you an account of my process of cleansing the canal.

Dr. Custer.—I don't think you entirely understood my question. I want to know how you wash the canals after using the preparation?

Dr. Schreier.—After using the preparation the canal is filled with a soapy matter as the result of the chemical action. I wash the canals with some weak solution of carbolic acid and water, so as to be sure not to carry into the canal any bacteria. For getting out all the particles of the soapy matter, I wrap a few fibers of cotton around a broach, dampen it with water, and then revolve it rapidly in the canal, because the soapy matter is soluble in water.

This procedure will quickly clean the canal.

Dr. A. H. Brockway, Brooklyn, N. Y.—I have had a little experience with this preparation, and I suppose that experience will be valuable to those who have not used it. Some weeks ago I got the preparation, and have used it in quite a number of cases, and I must say I am extremely pleased with the results. The question has been asked in regard to the danger of producing too great heat. I have not experienced any trouble from that, but in the first instance of using it I got a little pyrotechnic display by using too great a quantity and inadvertently getting a drop of water on it. It startled me, but the patient did not see it, because his mouth was covered with rubber-dam. The question has been asked, In what manner can the soapy contents of the canal be gotten rid of? My method is to use barbed instruments, but I mainly rely on hot water. Water being a solvent of the soap, the canal is thoroughly cleansed with it.

Dr. Reese.—I have used the preparation since May in about a dozen cases, and the patient did not know anything about the heat developed by the chemical action. In removing the soapy contents of the canal I have used peroxid of hydrogen; after drying the canal with cotton, I put in the treatment with some oil, and leave it there for a week, then I find the canals have a cleaner feeling than by using any other method.

Dr. Schreier.—It is of course very dangerous if you use too much of the preparation, because it is very explosive.

Dr. Reid, Chicago, Ill.—What is the proportion of sodium and potassium?

Dr. Schreier.—It is not a fixed quantity, but usually I use two parts of sodium and one of potassium, prepared in such a manner that it will adhere to the nerve-broach.

Dr. W. B. Ames, Chicago, Ill.—I would like to know when this preparation would be used. Would it be used when the pulp had been devitalized a day or two, or when the pulp was devitalized two or three weeks previously?

Dr. Schreier.—I treat it immediately after destroying the pulp, if there is insensibility. Any hemorrhage will be stopped by the introduction of the preparation.

Dr. Ames.—Would this combination of sodium and potassium act the same on the blood as the pulp?

Dr. Schreier.—It would.

Dr. Ames.—Is there much after-inflammation?

Dr. Schreier.—No; there is no soreness following.

Dr. Ames.—The reason why I ask this question is, I have adopted a treatment somewhat similar to this for treating putrescent pulp canals with nitrate of silver. When I want to get rid of the pulp-tissue in a canal, after using nitrate of silver, I always expect severe inflammation within a few days after the devitalization. This being a similar process, how often do you have inflammation?

Dr. Schreier.—With a sore tooth I proceed in the same manner as I do with the others.

Dr. Arnold, Columbus, Ohio.—If there had been absorption in the apex of the tooth, or if a blind abscess was present, would there be any difference in the process then?

Dr. Schreier.—I dont make any change in the treatment; I always find the fistulous openings closing after three or four days.

Dr. Florestan Aguilar, Cadiz, Spain.—I received a sample of it last September. I have used this preparation in all sorts of cases where the pulp had been devitalized for a long time, and also where the pulp had been devitalized only for two or three days, and I have used it in teeth which had abscesses, and my experience has almost always been satisfactory. In very few cases I have met with failure, but I did not blame the preparation; I think everybody has some failure in treating root-canals. I am very much pleased with the result of this preparation of sodium and potassium. I have gone a little further than Dr. Schreier in the employment of this preparation; he says he waits two or three days. I have had as good results by filling the canals immediately after cleaning them. I am always very careful in removing the rubber-dam, because if you let a little drop of the preparation fall on the cheek or gums it is apt to leave a mark on the face, which is very painful to the patient.

Dr. H. J. McKellops, St. Louis, Mo.—I am very much interested. Of course I have been many years in trying to save pulpless teeth. You can get into the upper root and the buccal root, but when you come to the anterior root what can you do? It is practice that shows me what these things amount to. When I started out to treat pulp-canals, we used creasote and I have a case of thirty-five years that is standing to-day; that tooth was treated and filled with creasote, and it was cured simply by that treatment. When you insert cotton in the canal with a broach, suppose you break the broach off. You have these things to contend with, and there is not one man in a thousand who can do all he says he can in treating these canals.

Dr. H. E. Beach, Clarksville.—I want to indorse a feature set forth by the essayist, the necessity for the destruction of all septic matter that is found in the tooth. His method is good We all know there is nothing that will destroy germs more readily than fire. It seems to me that this method which has been introduced by the essayist is easy. There are so many solvents of soap, that the best way to get the saponaceous matter out of the canal is to wash the canal out. If there are some particles left in the canal you can burn them up and wash out your canals with alcohol, then dry out the alcohol, and put in your filling.

Dr. Hinkins, Chicago, Ill.—I want to say a word ot caution about this compound, The caution is this, all sodium and potassium compounds are soluble in water. There is no better preparation to clean out this compound than water, as all sodium and potassium compounds are soluble in it.—*Cosmos.*

